# Therapeutic Strategies for Leukodystrophic Disorders Resulting from Perinatal Asphyxia: Focus on Myelinating Oligodendrocytes

**DOI:** 10.1007/s12035-017-0647-7

**Published:** 2017-06-28

**Authors:** Justyna Janowska, Joanna Sypecka

**Affiliations:** NeuroRepair Department, Mossakowski Medical Research Centre, Polish Academy of Sciences, 5 Pawinskiego str., 02-106 Warsaw, Poland

**Keywords:** Perinatal asphyxia, Neonatal hypoxia-ischemia, Oligodendrocyte progenitors, Myelinogenesis, Myelin structure, Electron microscopy, Neuroprotection, Cell-based therapies

## Abstract

Perinatal asphyxia results from the action of different risk factors like complications during pregnancy, preterm delivery, or long and difficult labor. Nowadays, it is still the leading cause of neonatal brain injury known as hypoxic-ischemic encephalopathy (HIE) and resulting neurological disorders. A temporal limitation of oxygen, glucose, and trophic factors supply results in alteration of neural cell differentiation and functioning and/or leads to their death. Among the affected cells are oligodendrocytes, responsible for myelinating the central nervous system (CNS) and formation of white matter. Therefore, one of the major consequences of the experienced HIE is leukodystrophic diseases resulting from oligodendrocyte deficiency or malfunctioning. The therapeutic strategies applied after perinatal asphyxia are aimed at reducing brain damage and promoting the endogenous neuroreparative mechanisms. In this review, we focus on the biology of oligodendrocytes and discuss present clinical treatments in the context of their efficiency in preserving white matter structure and preventing cognitive and behavioral deficits after perinatal asphyxia.

## Introduction

Perinatal asphyxia is the leading cause of neonatal brain injury known as hypoxic-ischemic encephalopathy (HIE). Accordingly, it is evoked by a temporarily limited (sometimes over a considerably prolonged period of time) supply of oxygen, which in turn leads to *hypoxia* or even anoxia in severe cases. The transiently reduced cerebral blood flow (*ischemia*) also results in the shortage of trophic support, especially the distribution of glucose. Perinatal asphyxia concerns about 4–6 of every 1000 full-term births and it is even more frequent in the case of children born prematurely [1]. Preterm delivery, which accounts for as much as 10% of newborns, as well as complications during labor are the major causes of birth asphyxia and make it to be the one of the leading causes of under-five child deaths [2–4]. Thanks to a constant progress in neonatal care programs, the mortality rate among newborn children has a tendency to decrease [5–7]. Nonetheless, the experienced deficiencies of oxygen and trophic support very often affect various body organs, including brain and trigger long-time consequences influencing the quality of life. Those include neurodevelopmental (neuromotor disorders, seizures, limb paresis) as well as cognitive and behavioral impairments [8–11].

Unfortunately, the cells constituting the neonatal nervous tissue are extremely sensitive to alterations in local homeostasis. In the perinatal period, a huge amount of neural progenitors arises in the effect of intense processes of neurogenesis and gliogenesis, contributing to the development of the central nervous system (CNS). The newly born neuroblasts give rise to specialized neurons like motoneurons, sensory neurons, or interneurons, which are responsible for behavioral and cognitive functions, as well as the interaction between those cells, respectively [12–14]. The physiological functioning of neurons is based on fast and efficient processing and transmitting signals within nervous system; therefore, any alterations usually lead to neurological disorders, which are pronounced to different extend. The process of signal transduction is highly energy-consuming and therefore neurons are supported by glial cells, which are present in at least an equal proportion to neurons, depending on a given brain region [15–17]. To enable salutatory conduction which is an efficient way of speeding-up propagation of impulses, axons are wrapped with myelin, elaborated by oligodendrocytes, the specialized glial cells.

## Alterations in Oligodendrocyte Development

**Fig. 1:**
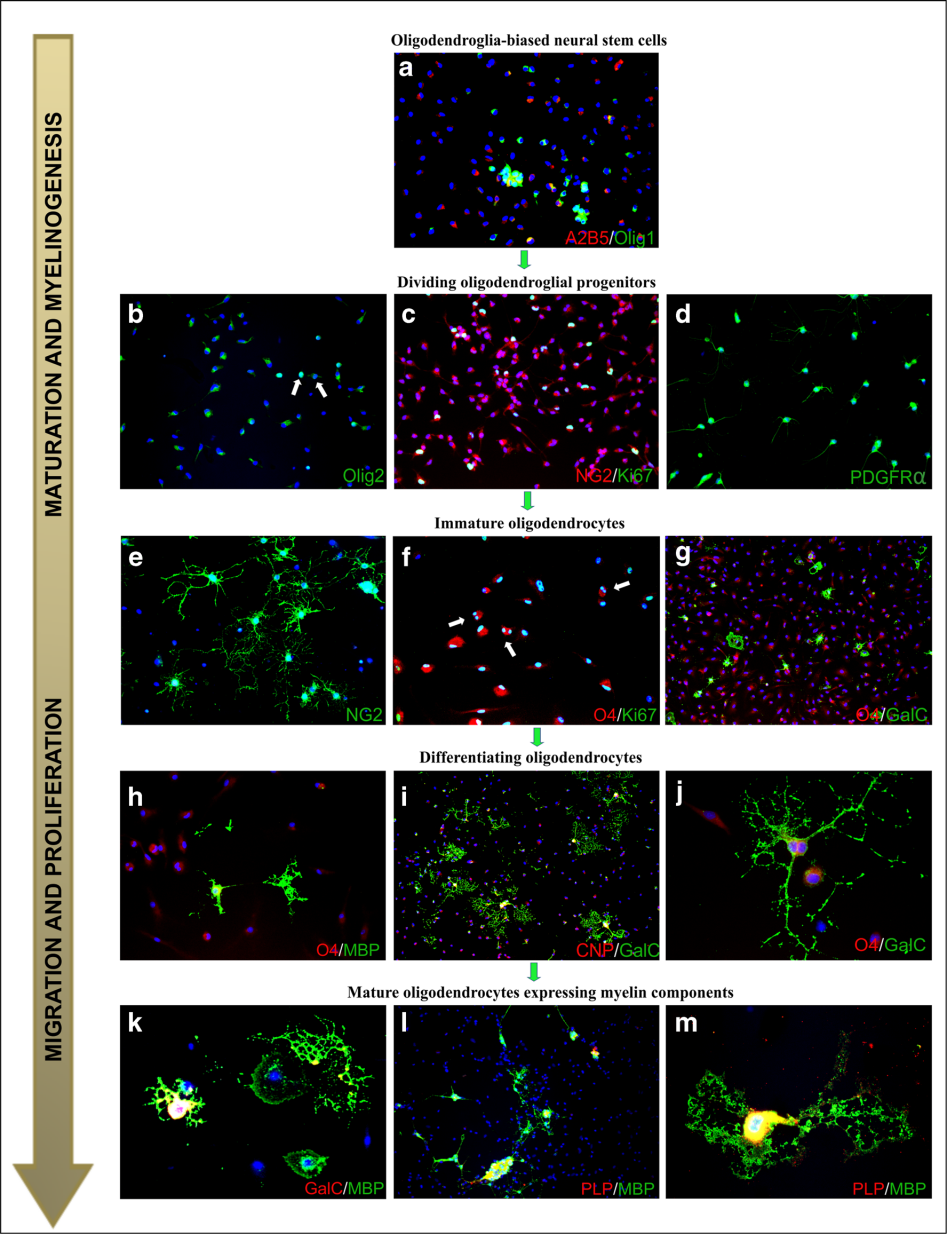
Differentiation of rat oligodendrocytes in a primary culture. Cell nuclei are stained with Hoechst 33258 (*blue*). **a** Neural stem cells, clearly discernible due to the expression of A_2_B_5_ marker (*red*), which are already oligodendroglia-biased (Olig-1 marker, *green*). **b** Oligodendroglial progenitors characterized by either nuclear (*arrows*) or cytosolic presence of transcription factor Olig-2 (*green*) after 24 h of in vitro culture. **c** Dividing OPCs expressing NG2 (*red*) and Ki67 (*green*) markers, indicating proliferating cells. **d** Visualization of PDGF-AA receptor (PDGFαR, *green*) characteristic for OPCs. **e** Immature NG2-positive cells (*green*), which are characterized by branched cell processes (*polydendrocytes*). **f** Immature oligodendrocytes recognized by their typical marker O4 (*red*), which are still able to divide, as indicated by Ki67 staining (*green*). **g** After 48 h of in vitro culturing, differentiating O4^+^ (*red*) oligodendrocytes express the GalC antigen (*green*). **h** The next step of oligodendrocyte (GalC^+^, *red*) differentiation associated with the expression of myelin components (MBP^+^, *green*). **i** Maturating cells recognized by their two most characteristic markers: CNP (*red*) and GalC (*green*). **j** Vanishing O4 presence (*red*) is replaced by GalC (*green*) expression in multibranched cells with long cellular extensions. **k** Cells with complex morphology, characterized by the presence of GalC (*red*) and MBP (*green*). **l** Mature oligodendroroglia expressing major myelin proteins: PLP (*red*) and MBP (*green*). **m** Magnification of double-labeled differentiated (PLP-red, MBP-green) myelinating oligodendrocyte on day 5 of in vitro culture

Myelin, which insulates axons and facilitates signal conduction, is essentially a compact multilamellar and highly organized structure [18, 19]. Generally, it is an extended and modified plasma membrane of oligodendrocytes, which are the cells responsible for myelinating CNS. Their precursors arise from neuroectoderma and populate the developing nervous system starting from approximately mid-gestation [20–22]. Gliogenesis is known to peak at the perinatal period and it proceeds intensely during the first postnatal months, in the course of constant neurogenesis. However, to gain capability for myelinogenesis, oligodendrocytes undergo a multistep process of maturation, which could be described by the expression of overlapping cell-specific markers (Fig. 1). Accordingly, the glial commitment of the neural stem cells is associated with the presence on their surface, the A2B5 marker (Fig. 1a), corresponding to ganglioside GT3 and its *O*-acetylated derivative epitope [23]. Oligodendrocyte progenitors cells (OPCs) (Fig. 1c–d) are commonly distinguished by the expression of transmembrane chondroitin sulfate proteoglycans (also known as NG2: neuron-glial antigen 2), which has been shown to be engaged in the cell migration and response to pathological signals [24–26]. The oligodendroglia-biased progenitors could be also distinguished by the expression of lineage-specific transcription factors Nkx2.2 and Olig2 [27–29]. Different localization of Olig1/Olig2 (either nuclear or cytosolic) is associated with the regulation of myelin genes and—after phosphorylation and acetylation—in process outgrowth [30, 31]. Interestingly, a significant number of NG2-positive cells, sometimes with already well-elaborated cell processes and therefore identified as *polydendrocytes* (Fig. 1e) [32, 33], remains in their undifferentiated state and is scattered in the brain parenchyma, both in the white and the gray matter [34]. They demonstrate a high proliferative potential and were shown to be the major population of cycling cells within the CNS [35–37].

OPCs however are the ultimate precursors of myelinating cells. The progress is their differentiation is associated with the appearance on the cell surface, the O4 and O1 markers (Fig. 1f–h), which are sulfatides attributed to immature cells, often termed *pre-oligodendrocytes* [38–41]. More advanced stages of oligodendrocyte maturation could also be identified by the intracellular presence of 2′,3′-cyclic nucleotide-3′-phosphodiesterase of CNPase (Fig. 1i), an enzyme engaged in myelin synthesis and maintenance [42, 43]. Differentiated cells are stained with a common marker against galactosylceramidase (GalC) (Fig. 1i–k), an enzyme hydrolyzing certain galactolipids, which are integrative myelin molecules [44–46]. Initiating myelin component synthesis opens up a new opportunity for immunostaining the mature oligodendrocytes. The antibodies against myelin basic protein (MBP), proteolipid protein (PLP) (Fig. 1k–m), myelin-associated glycoprotein (MAG), and myelin oligodendrocyte glycoprotein (MOG) are commonly used to visualize both the cells and the formed myelin sheaths [47–52]. Gained ability for myelinogenesis is the endpoint of oligodendrocyte maturation.

However, differentiation of progenitors into oligodendrocytes with extended and branched cell processes is an energy-consuming process. It requires constant trophic support to sustain the maturation process; otherwise, it could be inefficient or even arrested. As mentioned above, capability for myelinogenesis is associated with the activation of the set of genes coding for specific protein and lipid components and elaboration of several layers of lipid-rich layers of tightly compacted membrane. Poor vasculature or temporal limitation of oxygen and metabolic substrates due to reduced blood pressure might result in transient hypoxia, subsequently leading to activating the Wnt signaling pathway by upregulating the expression of hypoxia-inducible factors (HIFs) in oligodendrocytes [53]. Accordingly, in physiological oxygen level (also termed as “physioxia” and corresponding to 2–5% in nervous tissue), perinatal OPC-expressed HIF arrests cell differentiation yet promoting angiogenesis through Wnt7 signaling pathway in a paracrine manner. Once the microvasculature is established, HIF1α and HIF2α are deactivated by specific oxygen-dependent enzymes (asparaginyl and prolyl hydroxylases) and oligodendrocyte differentiation proceeds.

There is a growing list of evidence that oligodendrocytes are extremely sensitive to the alteration in local homoestasis and they respond to various kinds of pathological signals by either limited survival and arrested maturation process [54–57] or by increasing their proliferation rate and migrating towards the site of injury [37, 58, 59].

## Myelination of CNS by Mature Oligodendrocytes

Competed oligodendrocyte maturation means acquiring an ability to express myelin components, their intracellular transport, and incorporation into forming myelin sheaths. Although myelin is an extension of oligodendroglial cell membrane, its composition is highly modified in context of protein to lipid proportion. Whereas in cell membrane, this ratio is approximately 1:1, in myelin lipids constitute up to 70–85% lipids of dry mass, comprising predominantly cholesterol, galactosylceramide, and ethanolamine plasmalogen, which enable the close packing and tight organization of molecules within the membrane [60, 61]. Thus, numerous genes have to be activated (some of them are regulated by HIFs and therefore depend on the local level of oxygen) to express specific myelin components. The processes of membrane modification and generation have to be orchestrated and very efficient since large myelin quantities are elaborated by a given oligodendrocyte. In the CNS, almost all axons with diameters greater than 0.2 μm are myelinated and a large myelinated axon may have up to 250 to 300 turns of myelin wrapping around it [62]. Accordingly, the ratio between axon diameter and that of the total nerve fiber (axon and myelin) has been established to be about 0.6–0.7. Moreover, one oligodendroglial cell is responsible for myelinating several axons (making even 40 myelin segments) and is able to produce as much as 5–50 × 10^3^ μm^2^ of membrane a day [63]. Once established, myelin sheath is maintained by oligodendrocytes throughout adulthood, contributing to accelerating signal transduction even about 50–100-fold (up to 70–120 m/s for axons with diameter about 2–20 μm) [62].

Myelinated axons, besides being able to efficiently and rapidly propagate signals, are also protected by myelin from exogenous noxious stimuli. Neurodegenerative disorders developing in a consequence of CNS hypo/demyelination are actually based on unmyelinated axon dystrophy and their malfunctioning. A lesson learned from animal models point to the wide spectrum of neurological symptoms resulting from insufficient CNS myelination. They are pronounced to different extend, strongly depending on the severity of CNS dys/demyelination and include among others body tremor, focal sensory loss, limb paresis, and ataxia. Taking into consideration that during evolution, the amount of white matter tremendously increased achieving in primates and humans about 60% of brain volume (versus about 10% in rodents) [15], precise and efficient myelination seems to be crucial for correct CNS functioning.

## Leukodystrophic Disorders Resulting from Perinatal Asphyxia

**Fig. 2:**
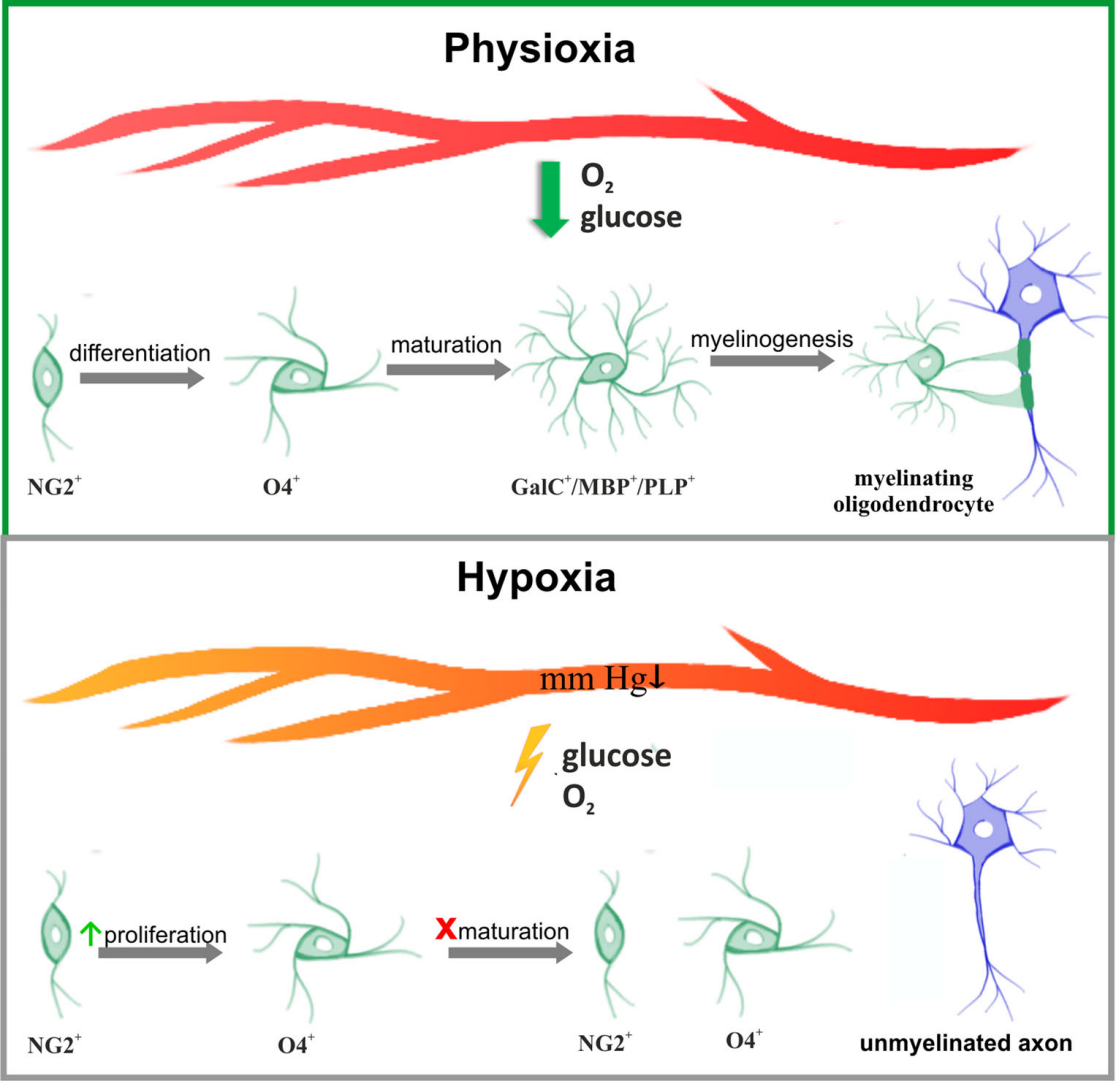
Impact of temporal hypoxia on the biology of oligodendrocytes. The processes of oligodendroglial differentiation, maturation and the capability for myelinogenesis are highly energy-consuming and are supported by metabolites provided by circulating, oxygenated blood (*upper panel*). Perinatal asphyxia leads to a decrease in blood pressure and a temporal limitation in oxygen and glucose supply (*lower panel*). Maturation of oligodendrocytes is arrested and myelinogenesis is altered/delayed

Delayed and/or disturbed maturation of oligodendrocytes, triggered by temporal limitation of oxygen and trophic support due to perinatal hypoxic-ischemic event, results in CNS hypomyelination and contributes to development of leukodystrophic diseases (Fig. 2). Since pathological insults affect various brain regions to a different extent, also the functions of oligodendrocytes would be either retained or altered and white matter lesions are diffused. The local intensity of inflammatory processes and the density of an extant vasculature have a significant impact on oligodendrocyte maturation and their efficiency in properly assembling myelin layers. It has been reported that endothelial cells associated with vessels play an important role in promoting the proliferation and survival of oligodendrocytes by secreting trophic stimuli to local microenvironment, defined as “oligovascular niche” [64–66]. Moreover, the enhanced vascularization process observed after stroke promotes oligodendrocyte survival and maturation—areas of the highest vessel density were also characterized by greater number of differentiated oligodendrocytes—suggesting a crucial role of constant trophic supply in physiological maturation of oligodendrocytes [67, 68]. Trophic coupling between endothelium and oligodendrocytes had been shown to contribute to maintaining the brain-blood barrier integrity [69]. Altogether, angiogenesis supporting cell survival, proliferation, and paracrine activity seems to be part of a strategic response to pathological clues leading to white matter injury.

**Fig. 3:**
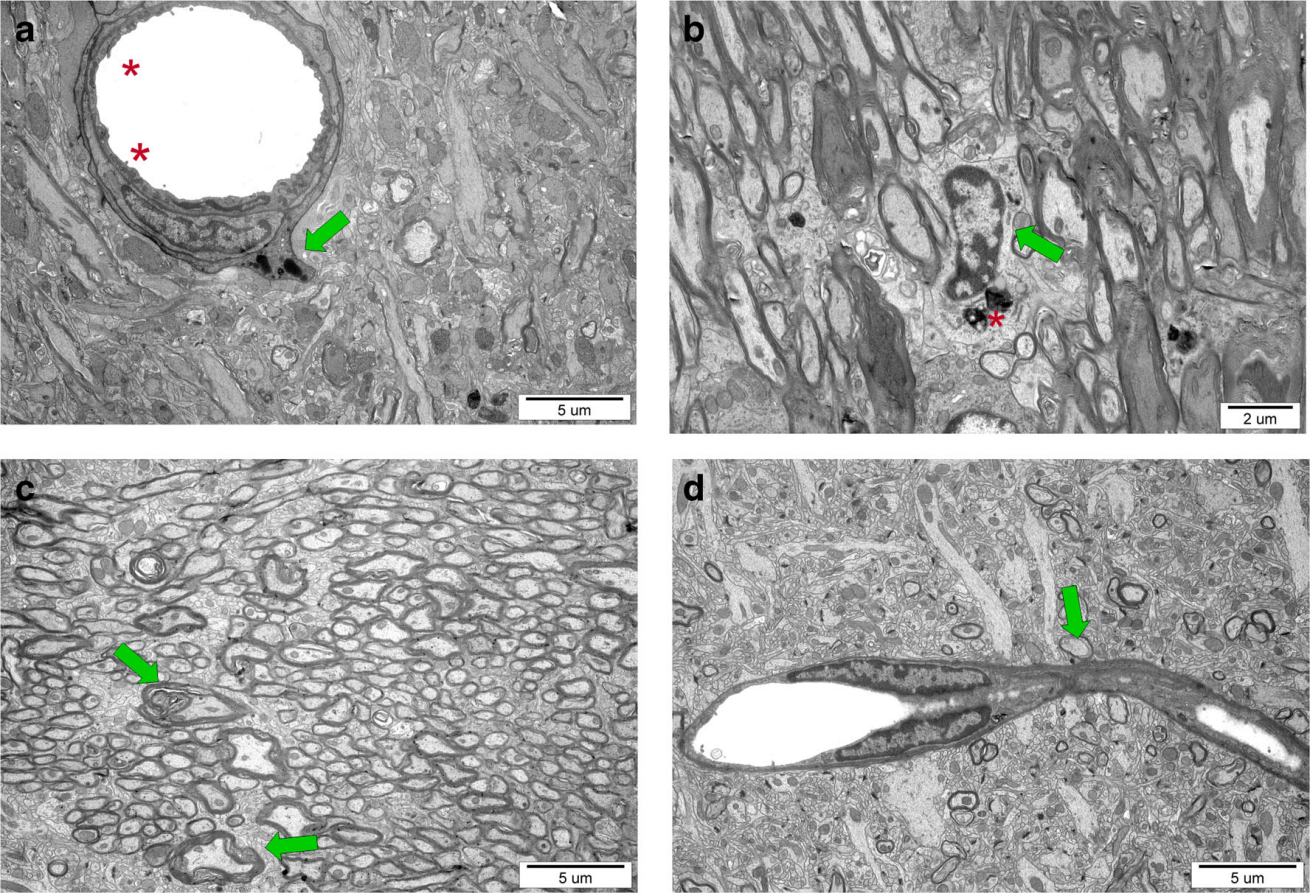
Ultrastructure of nervous tissue obtained from control and experimental rat brains 7 weeks after perinatal asphyxia performed in 7-day-old rat. **a** Corpus callosum of H-I rat: characteristic microvilli on the endothelium surface (*red asterisk*) and a macrophage cell residing in the blood vessel wall (*green arrow*) suggesting temporal interruption of blood-brain barrier. **b** Activated microglial cells (*green arrow*) with numerous lysosomes (*asterisk*), filled with hydrolytic enzymes, in the hippocampus of H-I rats. **c** Malformed myelin sheaths with splitting lamellae in striatum of injured rats. **d** The bridging vessel (*green arrow*) in H-I rats indicating the ongoing angiogenesis, conducive to processes of neurorestoration

In our in vivo studies on impact of perinatal asphyxia on oligodendrocyte survival and maturation in rat model, pathological changes in nervous tissue of various brain regions (hippocampus, striatum, corpus callosum, cerebral cortex) were observed even several weeks post insult. Accordingly, the ultrastructural examination by means of electron microscopy revealed many symptoms of locally ongoing inflammatory process like neuropil edema, collapsed small blood vessels, and macrophage infiltration (Fig. 3). However, the angiogenesis in the traumatized tissue was also detected, usually as the bridging vessels. The areas significantly depleted from mature oligodendrocytes were notified as well, which might correspond to the development of the diffuse white matter injury (DWMI)—one of the most characteristic outcomes of the HI episode [57, 70, 71].

Apart from limitation of trophic support and development of inflammatory process, the oxidative stress is thought to play a pivotal role in oligodendrocyte survival and differentiation after the hypoxic-ischemic insult. Increase in the level of free radicals (molecules containing an unpaired electron in an atomic orbital), especially the reactive oxygen species (ROS) and the reactive nitrogen species (RNS), strongly affects cell functioning and leads to the imbalance in local homeostasis. While at the physiological levels, ROS and RNS, generated during adenosine triphosphate (ATP) production by mitochondria, contribute to regulation of the cell survival and proliferation; their excess exerts a negative impact, especially by directly impairing mitochondrial function and often leading to an apoptotic cell death [72, 73].

In this way, oxidative stress is particularly harmful to oligodendrocytes due to their high metabolic demands and mitochondrial activity associated with generation and maintenance of myelin membranes. Since the process is energy dependent, significant amounts of ATP and oxygen are utilized, contributing to the increase in the ROS and RNS levels due to intense metabolism. Additionally, the high iron content, characteristic for oligodendrocytes and necessary for myelinogenesis, might contribute to generation of free radicals and subsequent lipid peroxidation. Free radical formation is also enhanced by cytokines associated with inflammatory process, which in reciprocal manner might enhance the ongoing inflammation, since hydrogen peroxide and ROS are used by the immune system as cytotoxic mediators [74]. It has been shown that pro-inflammatory cytokine-mediated downregulation of myelin genes is redox sensitive [75]. Free radicals might also participate in disrupting oligodendrocyte maturation by upregulating the expression of differentiation-inhibiting genes (like ID2, ID4) and downregulating the expression of genes promoting oligodendrocyte maturation (Sox10, Olig1, Olig2), by epigenetic mechanisms involving histone acetylation (repression of gene coding histone deacetylase 3, HDAC3) [76].

Physiologically, the detrimental effects of free radicals are prevented by several enzymes of antioxidant defense comprising superoxide dismutase-1 and dismutase-2 (SOD), catalase (CAT), glutathione peroxidase (GPx), and glutathione-S-transferase (GST). Oligodendrocytes, especially in the developing brain, are however characterized by the low concentrations of glutathione and SODs which would prevent either oxidative or nitrosative injury [77, 78]. Thus, supplementation with antioxidants which act as free radical scavengers seems to be indispensable for sustaining oligodendrogial functions after perinatal asphyxia.

Development of oxidative stress as a consequence of the experienced incident of perinatal asphyxia and its potential severity might be evaluated by determination of different biochemical markers, which usually are the products of reactions triggered by the excess of free radicals. While some of them are useful as indicators of lipid peroxidation (which also indirectly might provide information about myelin damage, like the presence of F2-dihomo-isoprostanes) [79], others correspond to either DNA or protein oxidative damage [80]. Reliable parameters, measured in the biologic fluids (plasma, urine, spinal fluid) and tissues, include relative concentration of isoprostanes (F2-IsoPs, F3-IsoPs), neuroprostanes (F4-NeuroPs), nonprotein-bound iron (NPBI), protein adducts of 4-hydroxynonenal (4-HNE PAs), and advanced oxidation protein products (AOPP), as well as the calculated ratio of reduced glutathione (GSH) to oxidized glutathione (GSSG), which might be considered as either predictive or discriminative indicator of oxidative stress [81–84].

Regional lack or malformation of myelin sheaths corresponding to CNS dys/demyelination unfortunately is not the only one outcome of deficiency of the mature oligodendrocytes. Beyond their major role of ensheathing axons of large and medium caliber [85], oligodendrocytes also were shown to support the nervous cells with energetic substrates like glycogen-derived pyruvate/lactate via the monocarboxylate transporter 1 [86–88] and with trophic factors [89]. The latter include factors promoting neurogenesis and protecting neurons after a temporal imbalance in tissue homeostasis. Thus, deficiency of either mature oligodendrocytes or even their undifferentiated progenitors could negatively influence survival of stressed neurons or those newly born after the insult.

## Rescuing Infants from Neonatal Hypoxic-Ischemic Event

**Fig. 4:**
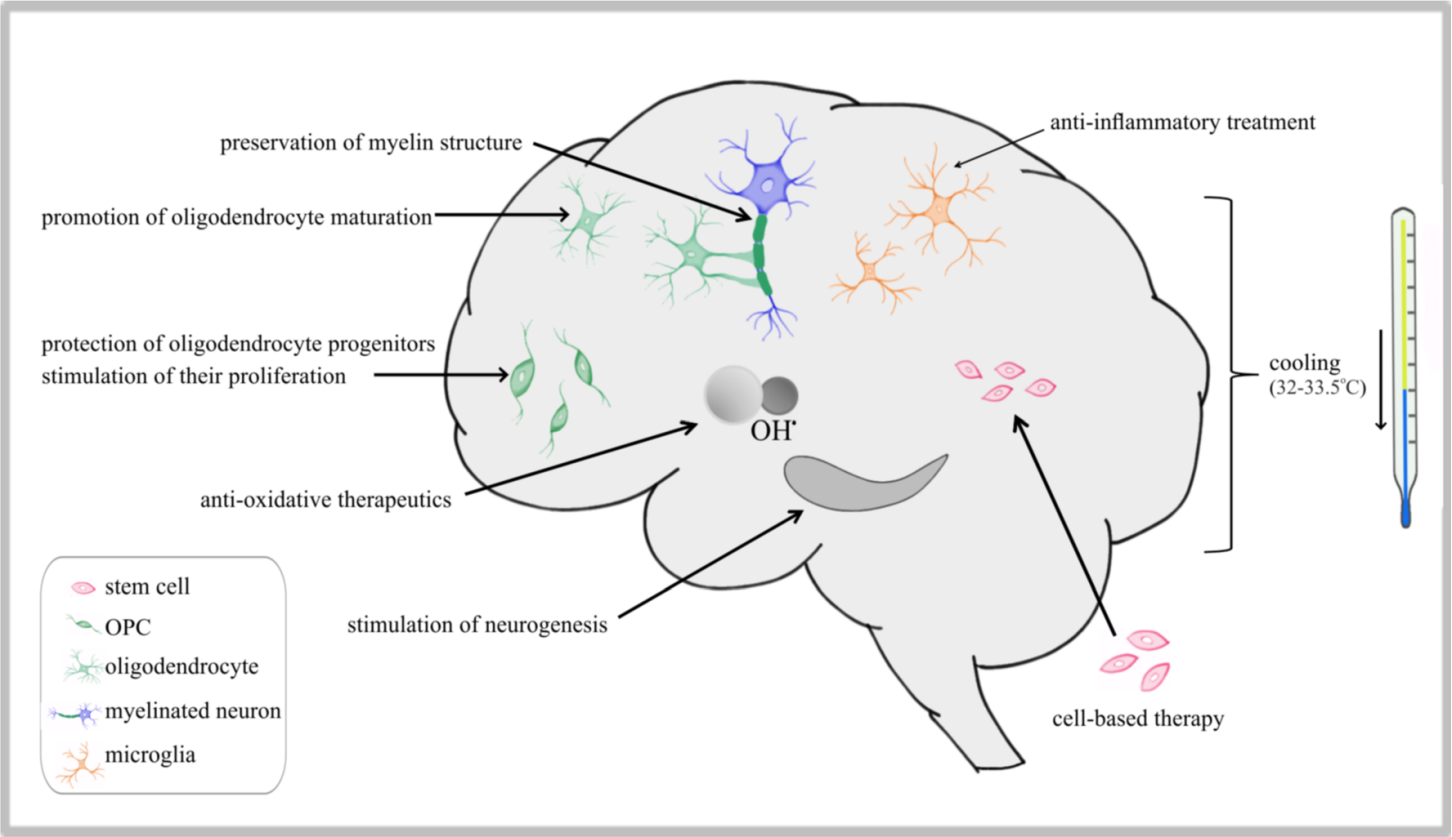
The scheme of targets for therapeutic intervention after perinatal asphyxia. The first and most effective is head cooling applied immediately after the injury, aimed at avoiding/limiting injures resulting from temporal hypoxia-ischemia. Current strategies are based on preventing the development of leukodystrophic disorders (anti-oxidative, neuroprotective are myelin-preserving protocols) and favoring endogenous neuroreparative mechanisms (providing anti-inflammatory therapeutics and trophic support)

To prevent neurodevelopmental impairments, which are being anticipated as fatal consequences of perinatal asphyxia, different types of remedies have been designed and recommended for clinical implementation (Fig. 4). In clinical practice, the very first treatment applied to the baby who has experienced neonatal hypoxia-ischemia and is unable to breathe without assistance is mechanical ventilation to restore the physiological level of blood oxygenation. To attain this goal, it is also necessary to stabilize blood pressure and to avoid hyperoxygenation which could have severe detrimental effects like bronchopulmonary dysplasia (BPD) and retinopathy of prematurity [90, 91]. Accordingly, the ventilatory support of term infants should be carried out with air and in the case of preterm infants, either air or a low concentration of oxygen (about 30%) should be applied, avoiding large-volume inflations [92, 93]. At the time of or after resuscitation, the body temperature should be maintained between 36.5 and 37.5 °C to ensure infant survival; however, later on mild hypothermia—either selective (head) or systemic (body)—is considered effective therapy preventing the development of neurological dysfunctions. Hypothermia as treatment is recommended for rescuing infants with moderate to severe HIE and is usually based on maintaining body temperature at 33–33.5 °C for 72 h followed by slow and controlled rewarming [94–97]. The published meta-analysis study revealed that mild hypothermia is indeed efficacious in promoting patient survival and in reducing neurological impairments [98, 99].

It however still remains to be disclosed if hypothermia exerts any influence on protecting the already existing myelin structure and on generation of new compact myelin. On the one hand, it was reported that considerably mild cooling (to 32–33 °C for 48 h) is effective in preventing cell death of oligodendroglial precursors and promoting their differentiation in the in vitro model, as well as contributes to accelerating the rate of their proliferation and increasing the number of myelinated axons in vivo [100, 101]. On the other hand, the delayed hypothermia followed by either slow or rapid rewarming was shown to trigger the apoptosis of myelinating oligodendrocytes in the piglet model of HI encephalopathy [102]. It indicates that very detailed procedures should be elaborated in regard to severity of the asphyxic event, the induction and duration of hypothermia, and the schemes of gradual rewarming, as well as therapeutic window for clinical interventions should be precisely determined to gain the best therapeutic effects.

Nonetheless, the mild hypothermia routinely applied in the case of perinatal asphyxia seemed to at least decrease morbidity among neonates who had experienced a severe hypoxic-ischemic insult. Its beneficial outcomes supposedly would be even better, when hypothermia is combined with other treatments, like melatonin administration, treatment with hydrogen sulfide, or 45–50% argon inhalation to increase brain metabolism, what was tested a piglet model of perinatal asphyxia [103–105].

## Pharmacological Treatments to Prevent Developmental Disabilities

For the last two decades, a number of natural biological substances has been included in preclinical studies on animal models, as well as used as treatments in a clinical practice (Table 1). The first group consists of the analogues of physiological compounds, which are known to play important roles in various biological processes. One of them is melatonin (*N*-acetyl-5-methoxy tryptamine), tested in the context of its utility in preventing neurodevelopmental disabilities. This hormone, which secretion by the pineal gland is regulated by the circadian rhythm (light/dark cycle), is thought to help stabilize basic physiological parameters like body temperature and blood pressure. The expected favorable outcome of melatonin administration is associated however with the reported direct anti-oxidative, anti-apoptotic, and anti-inflammatory effects [106, 107]. The latter is thought to contribute to either the protection of myelin structure and/or promotion of its reconstruction, in spite of the lack of the influence on the reduction of cortical infarct volume resulting from brain injury, as deduced from the study on the rat model [108, 109]. The encouraging data concerning the neuroprotective and immunomodulatory features of melatonin resulted in including this hormone in clinical trials as one of the potential therapeutic means to treat perinatal asphyxia.

**Table 1:** Currently used treatments administrated to new born children who experienced perinatal asphyxia

Biological factor	In vivo function	Natural source
Docosahexaenoic acid (DHA)—a long-chain omega-3 fatty acid	Sustains membrane fluidity and integrity; contributes to the synaptic functioning; an anti-inflammatory compound	Maternal milk during breast-feeding period; sea fish (like tuna, salmon, herring, sardines) caviar, algae
Resveratrol (3,5,4′-trihydroxy-trans-stilbene)	Enhances endogenous anti-oxidative defense	Skin of grapes, blueberries, raspberries, red wine, peanuts, dark chocolate
Sodium butyrate	Histone deacetylase inhibitor, regulates gene expression through NF-kappaB cascade; reduces expression of pro-inflammatory cytokines, stimulates neurogenesis; protects oligodendrocytes	Produced from dietary fiber in the gut by endogenous bacteria as the end-product of intestinal microbial fermentation; milk fat (so also in butter and cheese)
Erythropoietin (EPO, hematopoietin)	Indispensable for erythropoiesis, enhance angiogenesis, exerts neuroregenerative, anti-inflammatory and anti-apoptotic effects	Hormone produced by interstitial fibroblasts in the kidney
Melatonin (*N*-acetyl-5-methoxy tryptamine)	Direct anti-oxidative, anti-apoptotic and anti-inflammatory effects; protects myelin structure	Hormone secreted predominantly by pineal gland
Triiodothyronine (T3) and its prohormone, thyroxine (T4)	Engaged in physiological process of oligodendrocyte maturation; promotes in vivo remyelination	Hormones produced by the thyroid gland
Mesenchymal stem cells (MSCs)	Used for cell replacement, provide trophic support to diseased tissue, exert anti-inflammatory and neuroprotective effect	Bone marrow, umbilical cord (cord blood and Wharton’s jelly), adipose tissue, etc.

Another example of a physiological molecule is erythropoietin (EPO, hematopoietin), a pleiotropic cytokine predominantly produced in kidneys and liver and responsible for erythropoiesis [110]. By binding to its specific membrane receptor EpoR, EPO promotes proliferation and differentiation of erythroid progenitor cell. Importantly, this cytokine expression is upregulated in response to hypoxia [111]. Moreover, EpoR is found on different cell types in brain [112]. EPO was shown to enhance angiogenesis after anoxic event [113–115] and was suggested to have neuroregenerative, anti-inflammatory, and anti-apoptotic effects in the brain [116, 117]. It turned out to be effective in reducing to some extend white matter damage after HI injury in neonatal rats and preterm fetal sheep and in improving behavioral outcomes [118, 119]. Since nowadays EPO is routinely used in clinical practice to avoid or treat neonatal anemia [120], the additional advantages in preventing eventual neurological complications common in preterm neonates might supposedly be expected as well (phase 3 of clinical trials) [121–123].

According to the recently published data, magnesium sulfate was shown to accelerate the differentiation process of rat oligodendrocytes in vitro, although it did not ensure protection for mature cells against hypoxic-ischemic damage [124, 125]. While considering possible strategies aimed at rescuing oligodendrocyte progenitors from cell death and/or promoting their maturation, application of thyroid hormones should be taken into account. Triiodothyronine (T3) and its prohormone, thyroxine (T4), produced by the thyroid gland, are known to be engaged in physiological process of oligodendrocyte maturation and to promote remyelination in vivo [126, 127].

Another group of natural molecules comprising popular nutrients used to diminish fatal consequences of neurorodegenerative processes evoked during the hypoxic-ischemic episode could be distinguished. One of the potential remedies alleviating neurological deficits resulting from the asphyxic event is the docosahexaenoic acid (DHA), which is a long-chain omega-3 fatty acid (long-chain polyunsaturated fatty acids: LCPUFA) and yet the major polyunsaturated fatty acid in the adult mammalian brain. This phospholipid is responsible for membrane organization and integrity, exerts an impact on neurogenesis and neuroplasticity, contributes to modulating signal transduction pathways, participates in the myelination process, and affects the effectiveness of neurotransmission [128–131]. It has also been reported to possess anti-inflammatory properties and to participate in the development of the immune system in childhood [132–135]. Being crucial for a developing brain, DHA can be found in maternal milk during a lactation period (up to 1% of the total fatty acids) [136–138]. Supplementation with DHA was shown to be important for cognitive and visual development early in life and also useful for nervous system functioning during the life span [139–142]. Since LCPUFA, including DHA, can be easily found in seafood and certain plants [98, 143, 144], they have been recommended to be included in everyday, diversified diet. According to the very recent data, treatment with DHA turned out to be effective in the case of rat model of the neonatal hypoxia-ischemia. Accordingly, the beneficial effects of DHA administration seemed to result from both protecting neurons and myelin from destruction in inimical microenvironment and from reducing inflammatory response evoked by the HI episode. It was also shown to be effective in ameliorating cognitive deficits in rodent models [145, 146].

Resveratrol, a natural antioxidant found in grapes skin and red wine, is another example of neuroprotectant acting by enhancing endogenous anti-oxidative defense [147, 148]. Similar to the abovementioned molecules, it was shown to exert some beneficial effects on reducing cognitive impairments in the rodent model of stroke [149].

One of the worth mentioning molecules is also the sodium butyrate (SB), which is produced from dietary fiber in the gut by endogenous bacteria as the end-product of intestinal microbial fermentation. It acts as one of the histone deacetylase inhibitors (HDACs) and thus influences compaction of chromatin and regulates activation of genes engaged in progress of oligodendrocyte differentiation. HDACs may either initiate repression by modulating the acetylation state of nucleosomal histones/transcriptional regulators or directly bind to transcriptional regulators and function as transcriptional co-repressors [150]. SB has been also reported to possess immunomodulatory properties due to reducing the expression of pro-inflammatory cytokines [151–153]. In our recent studies on the rat model of neonatal hypoxia, the administration of SB was shown to exert neuroprotective, neurogenic, and anti-inflammatory effect finally resulting in a significant reduction of brain damage [154]. This hope-rising observation concerned prevention of the HI-induced loss of neuroblasts and oligodendrocyte precursor cells, which is important for the initiation of the compensatory mechanism leading to amelioration of neurological deficiency.

Keeping in mind the diversified and detrimental effects of perinatal asphyxia, new treatments are intensely searched for and preclinically tested in context of their eventual adverse effects and the desired effectiveness in preventing neurodevelopmental disorders. Pharmacological interventions are difficult in vulnerable newborns, since they might interfere with the intense developmental processes. Among proposed new treatments, administration of the free radical scavengers and anti-oxidative compounds seem to confer beneficiary effects in terms of enhancing natural, endogenous anti-oxidative defense, which is inefficient in early human development [78]. Catalpol, an iridoid glycoside extracted from Rehmannia root, has been shown to protect pre-myelinating oligodendrocytes through ERK1/2 signaling pathway by suppressing Ca2^+^ influx, reducing mitochondrial damage and inhibiting ROS overproduction [155]. Neuroprotective effects have been also achieved by administration of allopurinol (inhibitor of xanthine oxidase, the enzyme engaged in generation of superoxide particle), vitamin E (α-tocopherol and β-tocopherol) with vitamin C (ascorbic acid) together counteracting lipid peroxidation, as well as deferoxamine, which is chelating agent for free iron (highly concentrated in oligodendrocytes) [156, 157]. Nonetheless, the new effective therapies preventing the harmful effects of oxidative stress and promoting reparative processes are needed to be developed and preclinically tested to provide safe and efficient treatment options for newborns.

## Cell Transplantation as a Neuroreparative Strategy

A growing list of evidence from preclinical studies indicates that cell transplantation is an effective treatment option applied to prevent the symptoms of neurodegenerative processes which develop as a consequence of cell death or alteration in their differentiation and biological functions. Directly replenishing the nervous tissue with cells which are depleted in a result of the insult seems to be one of the main advantages of cell-based therapies. The mesenchymal stem cells obtained from various sources might be used either for direct engraftment with aim of their in vivo differentiation or for generation of neural progenitors. One of the most promising sources of mesenchymal stem cells is umbilical cord (cord blood and Wharton’s jelly) [158, 159]. In our very recent studies, human cord blood-derived cells were shown to be relatively easily differentiated into oligodendroglia-biased progenitors by application of the serum-free protocol based of using analogues of physiological molecules (PDGF-AA, T3, and extracellular matrix components like laminin and fibronectin) [160]. The obtained progenitors might in vivo give rise to oligodendrocyte with myelinating potential and/or to favorably act by modifying the local tissue microenvironment by secreting trophic factors and anti-inflammatory cytokines [89, 161].

Promoting neurological recovery via indirect bystander actions seems to be one of the main advantages of using cells with stem cell/progenitor characteristics [162, 163]. Accordingly, transplantation of the mesenchymal stem cells was shown to contribute to the reduction of the lesion volume and protection of white matter and consequently to improve the motor function [164–166]. Thus, the cell-based therapies offer few advantages: a direct supplementation of the traumatized tissue with the exogenous cells, protection against further brain damage, promotion of the neuroregeneration, and improvement of the behavioral functions.

## Clinical Perspective

When concluding on the therapeutic strategies for preventing leukodystrophic disorders resulting from perinatal asphyxia, it should be taken into consideration that neurons and glial cells are functionally interdependent. During development, oligodendrocyte maturation and myelinogenesis is guided by the external stimuli, including signals provided by differentiating neurons [167–169]. While some of them regulate the differentiation process (PDGF-A, neuregulin: NGR, etc.), others play a role in matching oligodendrocytes to the axonal surface (cell adhesion molecule L1, the polysialylated neuronal cell adhesion molecule: PSA-NCAM; Jagged 1). Similarly, neuron survival and proficient functioning strongly depends on oligodendrocytes and compact myelin sheath [170–173]. Keeping the above in mind, both the compounds protecting either neurons or oligodendrocytes, as well as those preserving the myelin structure, might turn out to be effective in preventing white matter damage and subsequent neurodevelopmental disorders. On the one hand, the common feature of the enumerated compounds is their relative fine tolerance by very young organisms, as deduced from very rare reports on side effects evoked by natural molecules administration. On the other hand, however, their efficacy of improving the overall well-being of human neonates, who experienced perinatal hypoxia, is still very limited. It results from a wide range of the injuries evoked by HI, the course of pregnancy (like potential chronic hypoxia, hemorrhages), the procedures initiated in response to the insult and time of their implementation, very limited number of clinical trials, and confined abilities of the already existing compounds to cure severe trauma. The solution for increasing the efficiency of successful treatment would be to foster preclinical studies on animal models with new substances leading to detailed description of mechanisms of their action and their eventual side effects on both molecular and systemic levels. The other one could be combining the already known treatments with the aim of increasing their beneficial effects, like for instance administration of neuroprotectants (sodium butyrate, melatonin, DHA) with cell therapies promoting endogenous repair. As deduced from the available data concerning the outcomes of the applied therapies (which turned out to be ineffective in some cases), in spite there is a growing list of the treatments available, the clinical protocol should be every time chosen individually for the given case of perinatal asphyxia event.
